# Melatonin Decreases Glucose Metabolism in Prostate Cancer Cells: A ^13^C Stable Isotope-Resolved Metabolomic Study

**DOI:** 10.3390/ijms18081620

**Published:** 2017-07-26

**Authors:** David Hevia, Pedro Gonzalez-Menendez, Mario Fernandez-Fernandez, Sergio Cueto, Pablo Rodriguez-Gonzalez, Jose I. Garcia-Alonso, Juan C. Mayo, Rosa M. Sainz

**Affiliations:** 1 Departamento de Morfologia y Biologia Celular, Instituto Universitario Oncologico del Principado de Asturias (IUOPA), Universidad de Oviedo, 33006 Oviedo, Spain; heviadavid@uniovi.es (D.H.); gonzalezmpedro@uniovi.es (P.G.-M.); 2 Departamento de Quimica Fisica y Analitica, Universidad de Oviedo, 33006 Oviedo, Spain; marioupl@hotmail.com (M.F.-F.); rodriguezpablo@uniovi.es (P.R.-G.); jiga@uniovi.es (J.I.G.-A.); 3 Servicios Cientifico Tecnicos, Edificio Severo Ochoa, Universidad de Oviedo, 33006 Oviedo, Spain; srgcueto@gmail.com

**Keywords:** melatonin, metabolism, ^13^C-glucose, lactate, ATP, prostate cancer

## Abstract

The pineal neuroindole melatonin exerts an exceptional variety of systemic functions. Some of them are exerted through its specific membrane receptors type 1 and type 2 (MT1 and MT2) while others are mediated by receptor-independent mechanisms. A potential transport of melatonin through facilitative glucose transporters (GLUT/*SLC2A*) was proposed in prostate cancer cells. The prostate cells have a particular metabolism that changes during tumor progression. During the first steps of carcinogenesis, oxidative phosphorylation is reactivated while the switch to the “Warburg effect” only occurs in advanced tumors and in the metastatic stage. Here, we investigated whether melatonin might change prostate cancer cell metabolism. To do so, ^13^C stable isotope-resolved metabolomics in androgen sensitive LNCaP and insensitive PC-3 prostate cancer cells were employed. In addition to metabolite ^13^C-labeling, ATP/AMP levels, and lactate dehydrogenase or pentose phosphate pathway activity were measured. Melatonin reduces lactate labeling in androgen-sensitive cells and it also lowers ^13^C-labeling of tricarboxylic acid cycle metabolites and ATP production. In addition, melatonin reduces lactate ^13^C-labeling in androgen insensitive prostate cancer cells. Results demonstrated that melatonin limits glycolysis as well as the tricarboxylic acid cycle and pentose phosphate pathway in prostate cancer cells, suggesting that the reduction of glucose uptake is a major target of the indole in this tumor type.

## 1. Introduction

Melatonin (*N*-acetyl-5-methoxy-tryptamine), the main product of the pineal gland in mammals, was found present in almost all life forms including bacteria, unicellular organisms, algae, plants, invertebrates, and vertebrates [1,2,3]. In addition to being an ancient molecule, in mammals and other vertebrates melatonin synthesis is not limited to the pineal gland but it has also been found in many other tissues and organs including retina, Harderian gland, gut mucosa, cerebellum, airway epithelium, liver, kidney, adrenals, thymus, thyroid, pancreas, ovary, carotid body, placenta, endometrium, mast cells, natural killer cells, eosinophilic leukocytes, platelets, and endothelial cells [4].

Classically, melatonin is well known as a neurohormone that regulates seasonal behavior and sleep in vertebrates. Its circadian synthesis and release make it a chemical signal that entrains the organism’s physiology into the light:dark period [5]. However, melatonin exerts an exceptional variety of systemic functions including the ability to regulate the circadian entrainment, sleep promotion, anti-inflammatory activity or immune enhancement [6,7]. At the cellular level, melatonin reduces proliferation, decreases cell death and exerts antioxidant properties [8,9,10].

Some actions exerted by melatonin are mediated through its specific membrane receptors type 1 and type 2 (MT1 and MT2) including anticonvulsant properties, its protective effect against myocardial infarction and the ability to inhibit the effects of estrogens [11,12,13,14]. However, others are mediated by receptor-independent mechanisms [10]. In this regard, the ability of the indole to scavenge reactive oxygen and nitrogen species (ROS/RNS) [15,16] or activate antioxidant enzymes has received special attention [17]. At both physiological and pharmacological concentrations, melatonin attenuates oxidative stress and regulates cellular metabolism by receptor independent mechanisms [18].

In addition to identify its target tissues, other issues have recently focused the attention about the indole, including the mechanism(s) by which melatonin is taken up by cells [19]. In fact, it was recently described that melatonin could cross cell membranes through glucose transporters (GLUT/*SLC2A*). A reduction of glucose uptake might be responsible of melatonin’s activity to reduce the growth and progression of prostate cancer cells [20]. Even though melatonin was found to reduce lactate production [21], it is still unknown how the indole could affect glycolysis in cancer cells.

Melatonin influences insulin secretion from pancreatic islets via MT1 and MT2 receptors [22]. In fact, melatonin administration to diabetic rats decreases blood glucose and increases fatty acids [23,24,25]. Furthermore, melatonin has beneficial effects in the prevention of hyperglycemia by modulating insulin release [26]. Some specific polymorphisms of melatonin receptors have been recently associated with an increased risk of type 2 diabetes mellitus [27,28].

Prostate cancer is the second leading cause of death of cancer in men in western countries. Although diagnostic tools such as prostate-specific antigen (PSA) levels have helped early detection of new cases of prostate cancer (PCa), there is no satisfactory therapy for advanced hormone-refractory tumors. Since PCa is primarily sensitive to androgens, initial strategies for prostate tumors include androgen ablation but unfortunately, throughout time (3–5 years), tumors become androgen independent. The disease clearly correlates with age and the prevalence of PCa is so high among aged males that it could be considered a normal age-related phenomenon. Melatonin reduces cell growth of both androgen sensitive and insensitive prostate cancer cells [29], and reduces tumor progression in a murine model by an insulin like growth factor protein 3 (IGFBP3) mediated mechanism [30].

Changes in cell metabolism along with cancer progression, have been recently considered a new hallmark of cancer cells [31,32]. The prostate has a particular metabolism that changes with tumor progression. In differentiated epithelial cells, citrate is the final product of glucose metabolism and prostate cells show very low levels of oxidative phosphorylation (OXPHOS) and an increase glycolytic rate [33]. However, during the first steps of carcinogenesis, OXPHOS is reactivated and cancer cells switch to the “Warburg effect” only during the metastatic or castration resistant stages.

Since melatonin reduces the growth of prostate tumors and it has been proposed that it influences glucose uptake by prostate cancer cells [19], we hypothesize that melatonin, by altering the uptake of glucose in prostate cancer cells, might change its metabolic switch between oxidative or glycolytic. In order to study this, we used a novel and sensitive methodology based on ^13^C stable isotope-resolved metabolomics as an experimental approach to evaluate the enrichment of metabolites in ^13^C in androgen sensitive LNCaP and insensitive PC-3 prostate cancer cells after melatonin incubation.

## 2. Results

### 2.1. Melatonin Decreases Glucose Uptake in Prostate Cancer Cells

**Figure 1:**
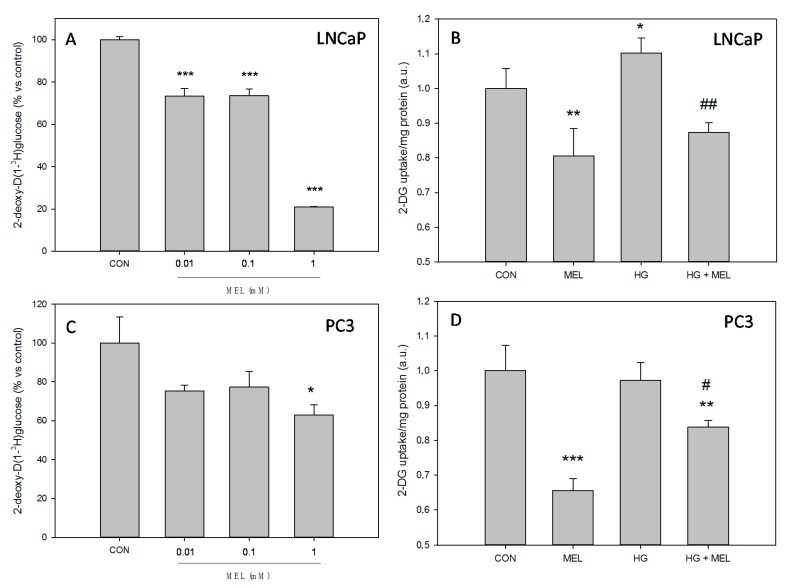
Melatonin reduces glucose uptake in prostate cancer cells. Labeled 2-deoxy-d-[1-^3^H]glucose uptake was performed in LNCaP cells (**A**); and PC-3 cells (**C**) after 10 min of incubation in the presence of increasing concentrations of melatonin. 2-deoxy-d-glucose (2-DG) uptake was performed in: LNCaP (**B**); and PC-3 cells (**D**) grown in media supplemented with 2 g/L glucose (CON) or 4 g/L glucose (HG) with or without 1 mM melatonin (MEL) for 24 h. Arbitrary 1.0 was given to CON groups. A representative of three independent experiments is shown. Results are represented as mean ± SEM (*n* = 3). * *p* < 0.05 vs. CON, ** *p* < 0.01 vs. CON, *** *p* < 0.001 vs. CON, ^#^ *p* < 0.05 vs. HG, ^##^ *p* < 0.01 vs. HG.

The role of melatonin on the glucose uptake was studied in both, androgen dependent LNCaP and independent PC-3 prostate cancer cells. First, a dose–response study was performed. Melatonin reduced significantly the levels of glucose uptake at all concentrations tested in LNCaP cells (Figure 1A). In addition, under normal (2 g/L) or high glucose (4 g/L) (HG) concentrations in the culture media, melatonin significantly reduced the uptake of glucose. As expected, HG favored a higher glucose uptake in LNCaP cells, suggesting a positive regulation and melatonin reduced it (Figure 1B). On the other hand, melatonin reduced the uptake of glucose at the highest concentration tested (Figure 1C). Curiously, a higher glucose concentration in culture media did not increase the glucose uptake in PC-3 cells (Figure 1D), contrary to what was observed in LNCaP, but melatonin significantly reduced the uptake when HG media was employed.

### 2.2. Melatonin Reduces ATP/AMP Ratio in Prostate Cancer Cells

**Figure 2:**
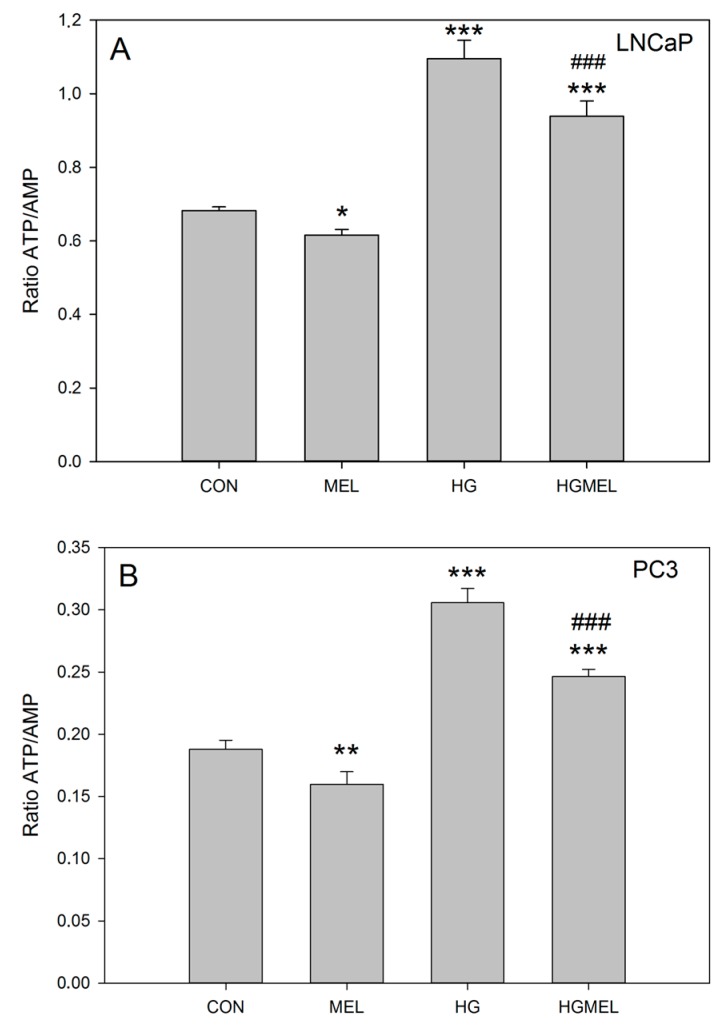
Melatonin decreases ATP/AMP balance in prostate cancer cells. ATP and AMP were measured by HPLC in: LNCaP (**A**); and PC-3 cells (**B**) grown in media supplemented with 2 g/L glucose (CON) or 4 g/L glucose (HG) with or without 1 mM melatonin (MEL) for 24 h. A representative experiment of three is shown. Results are represented as mean ± SEM (*n* = 3). * *p* < 0.05 vs. CON, ** *p* < 0.01 vs. CON, *** *p* < 0.001 vs. CON, ^###^ *p* < 0.001 vs. HG.

In order to evaluate whether the reduction of glucose uptake altered oxidative phosphorylation (OXPHOS) or glycolysis in prostate cancer cells, ATP levels were evaluated after melatonin incubation. For this purpose, ATP and AMP levels were quantified by HPLC after culturing cells with or without melatonin in normal or HG media. First, it was observed that, as expected, prostate cancer cells cultured in high glucose media (HG) produced higher levels of ATP (Figure 2), being this increase more significant in LNCaP than in androgen insensitive PC-3 cells. Melatonin significantly decreased ATP/AMP ratio by 10% when cells were cultured in normal glucose concentration but it was higher (roughly 20%) when cells were cultured in HG media in both cell types.

These results correlated with a lower glucose uptake in both cell lines and prompted us to study whether these changes could also be related to a reduction of tricarboxylic acid cycle (TCA) in both androgen sensitive and insensitive prostate cancer cells.

### 2.3. Increase Glucose Concentration Rise CO_2_ Released in Androgen Sensitive Prostate Cancer Cells

**Figure 3:**
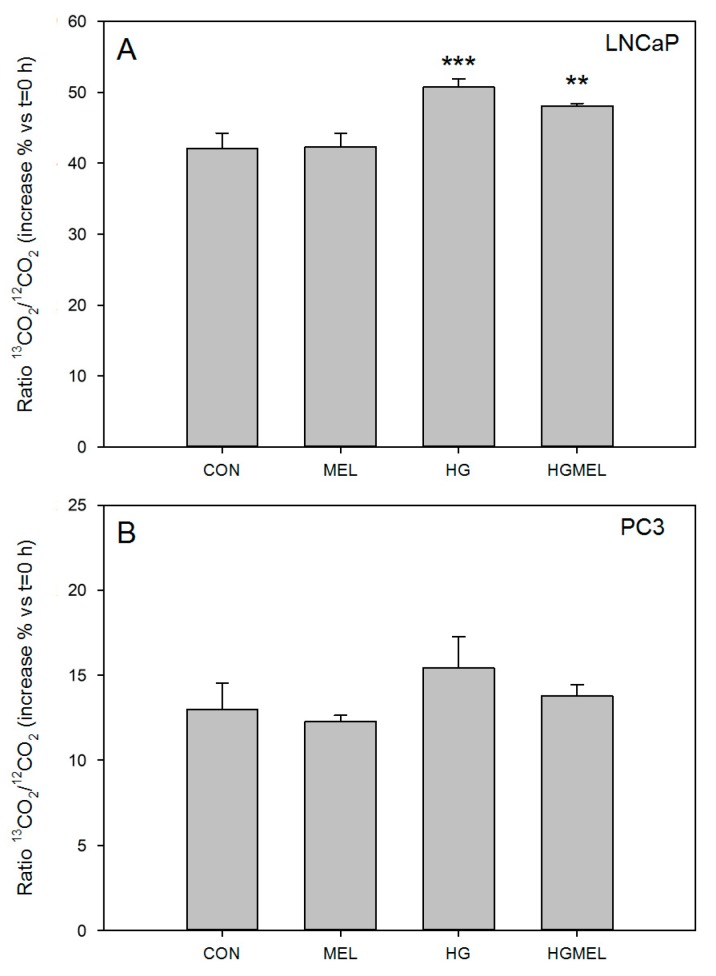
Changes CO_2_ production by prostate cancer cells. Cells were grown in the presence of ^13^CO_2_. ^13^CO_2_/^12^CO_2_ ratio was calculated in: LNCaP (**A**); and PC-3 (**B**) cells after treatment with or without 1 mM MEL after 6 h of culture media supplemented with 2 g/L of U-^13^C-glucose (CON) or 4 g/L of U-^13^C-glucose (HG). ^13^CO_2_ production was measured at the beginning of the experiment (Time = 0) and at the end of the experiment (Time = 6 h). Results are shown as the increment vs. T_0_. A representative experiment of three is shown. Data are represented as mean ± SEM (*n* = 3). ** *p* < 0.01 vs. CON, *** *p* < 0.001 vs. CON.

Several steps of glucose utilization are associated with the production of CO_2_. Then, it is expected that, if glucose uptake and ATP generation are reduced by melatonin in prostate cancer cells, CO_2_ released could also be affected. Thus, by using U-^13^C-d-glucose as the source of glucose and after measuring the ^13^CO_2_/^12^CO_2_ ratio produced by ^13^C isotopic enrichment with a GC-IRMS instrument, labeled CO_2_ that was produced and released in the presence or absence of melatonin was measured. As shown in Figure 3, there is an increase in ^13^CO_2_/^12^CO_2_ release when cells were cultured in HG medium, concomitant with the higher glucose uptake previously observed. Melatonin only limited the increase in ^13^CO_2_/^12^CO_2_ ratio when LNCaP cells were incubated in HG media. The decrease in CO_2_ production was lower than expected from differences found in glucose uptake or in ATP production.

### 2.4. Melatonin Decreases the Tricarboxylic Acid Cycle (TCA) in Androgen Dependent Prostate Cancer Cells and ^13^C-Lactate Labeling in Both Cell Types

**Figure 4:**
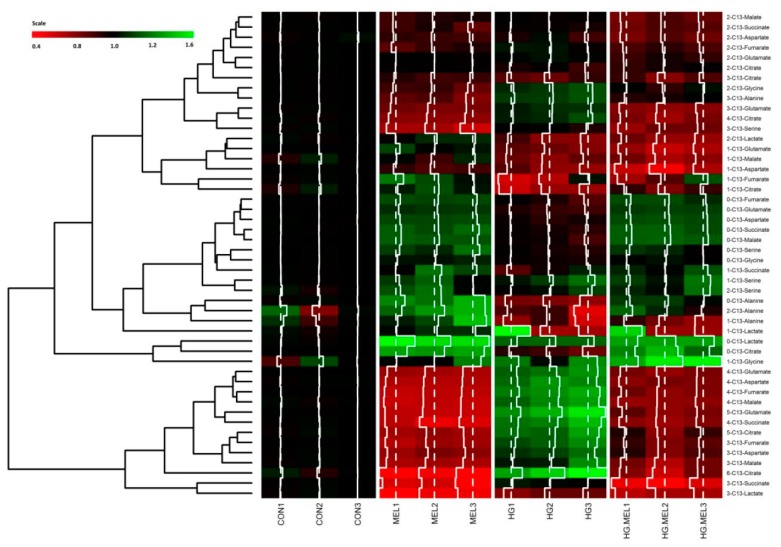
Effect of melatonin on the metabolic profile of LNCaP cells grown in normal or high glucose media. Heat map of log2 fold changes of tricarboxylic acid cycle (TCA) cycle metabolites after 24 h of culture with 2 g/L of U-^13^C-glucose (CON) or 4 g/L of U-^13^C-glucose (HG) in the presence or absence of melatonin (MEL) 1 mM. Values of labeled metabolites were normalized to CON group. A color scale from 0.4 (red) to 1.6 (green) was employed to represent the amount of each compound. Red compounds are metabolites with lower levels than CON and green compounds are metabolites with higher levels than CON. Annotations included on left side of the heat map show the clustering of the glucose metabolites studied.

Previous results pointed out that melatonin was able to decrease glucose metabolism in prostate cancer cells. Melatonin is well-known as an antioxidant and its role in mitochondria is reported elsewhere [34]. These encouraged us to study, by using a ^13^C-metabolomic approach, the molar fractions of 11 mitochondrial metabolites derived from glucose in LNCaP and PC-3 grown in the presence of ^13^C-labeled glucose. Metabolites heat map is represented in Figure 4. LNCaP cells showed a significant change of metabolic signature when grown in normal or HG culture media. Moreover, remarkable differences were noticed when androgen-dependent cells were cultured in the presence of melatonin, under either normal or HG conditions, when compared to parental, non-treated cells.

**Figure 5:**
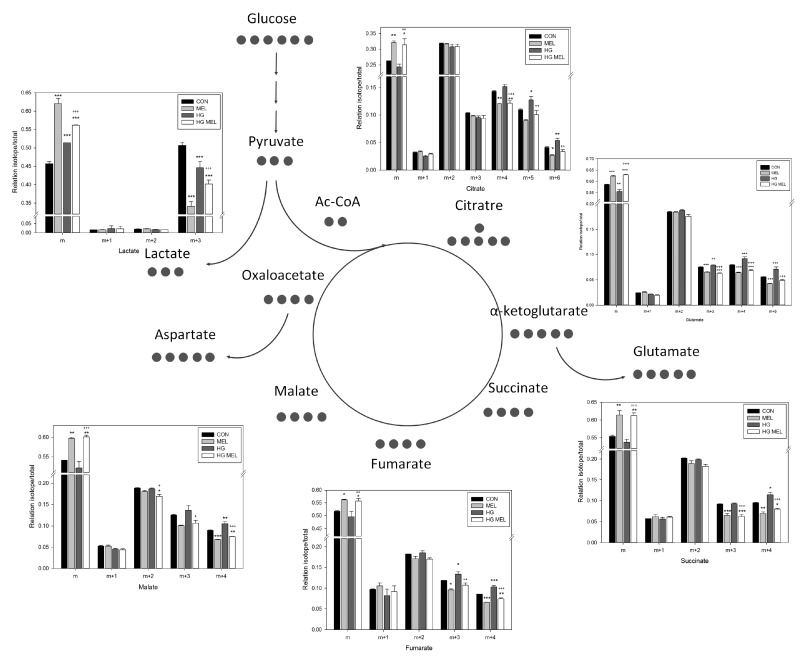
Schematic representation of tricarboxylic acid cycle (TCA) cycle. Black circles represent the number of labeled carbons of each compound. Graphs represented metabolites and their relation isotope/total in ^13^C among 2 g/L of U-^13^C-glucose (CON) or 4 g/L of U-^13^C-glucose (HG) in the presence or absence of melatonin (MEL) 1 mM. “m” represents unlabeled metabolite and “m + n”, labeled in “n” number of carbons. * *p* < 0.05 vs. CON, ** *p* < 0.01 vs. CON, *** *p* < 0.001 vs. CON, ^+^ *p* < 0.05 vs. HG, ^++^ *p* < 0.01 vs. HG, ^+++^ *p* < 0.001 vs. HG.

A complete ^13^C incorporation diagram (m, m + 1, and m + 6) in lactate, citrate, glutamate, succinate, fumarate or malate is shown in Figure 5. Melatonin decreased levels for all those ^13^C-labeled TCA metabolites when cultured in both normal and HG conditions. As it could be deduced, an inverse situation occurs when the non-labeled metabolites are analyzed in melatonin-incubated cells, since these metabolites are expressed as a percentage of the total amount of the compound. Among the metabolites studied, the highest difference among experimental groups was found in labeled lactate. Since all d-glucose carbons were ^13^C labeled, lactate production was dramatically reduced (close to 50%) in melatonin-treated cells. The effect was even higher than those found in other TCA metabolites (roughly 15% decrease) after 24 h of culture. The decrease caused by the indole was also found in HG media conditions, but the reduction was lower.

**Figure 6:**
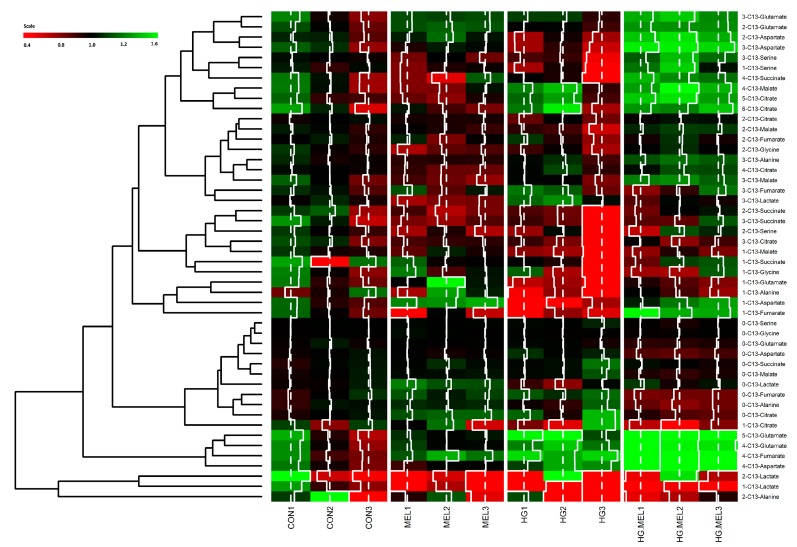
Effect of melatonin on the metabolic profile of PC-3 cells grown in normal or high glucose media. Heat map of log2 fold changes of tricarboxylic acid cycle (TCA) metabolites after 24 h of culture with 2 g/L of U-^13^C-glucose (CON) or 4 g/L of U-^13^C-glucose (HG) in the presence or absence of melatonin (MEL) 1 mM. Values of labeled metabolites were normalized to CON group. A color scale from 0.4 (red) to 1.6 (green) was employed to represent the amount of each compound. Red compounds are metabolites with lower levels than CON and green compounds are metabolites with higher levels than CON Annotations included on left side of the heat map show the clustering of the glucose metabolites studied.

**Figure 7:**
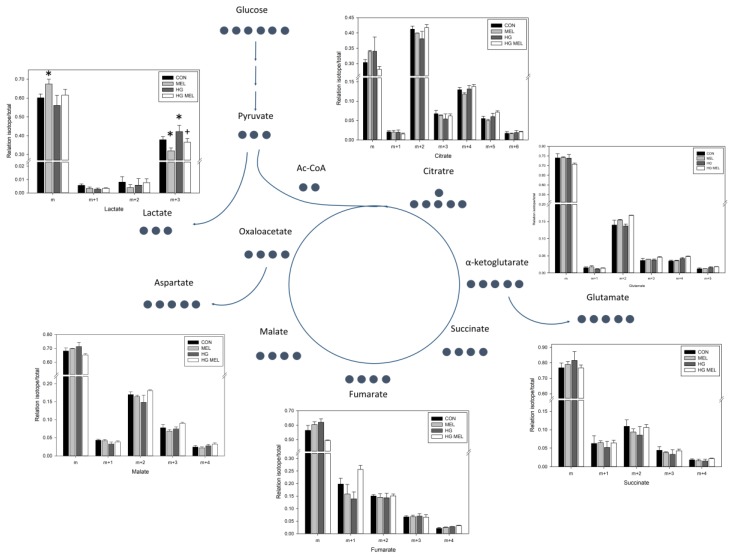
Schematic representation of tricarboxylic acid cycle (TCA). Black circles represent the number of labeled carbons of each compound. Graphs represented metabolites and their relation isotope/total in ^13^C among 2 g/L of U-^13^C-glucose (CON) or 4 g/L of U-^13^C-glucose (HG) in the presence or absence of melatonin (MEL) 1 mM. “m” represents unlabeled metabolite and “m + n”, labeled in “n” number of carbons. * *p* < 0.05 vs. CON and ^+^ *p* < 0.05 vs. HG

In the case of androgen-independent PC-3 cells, melatonin did not change any TCA metabolite studied, even when cells were culture in HG media (Figure 6). However, it reduced lactate ^13^C-labeling in these cells (Figure 7).

### 2.5. Melatonin Decreases LDH Activity

**Figure 8:**
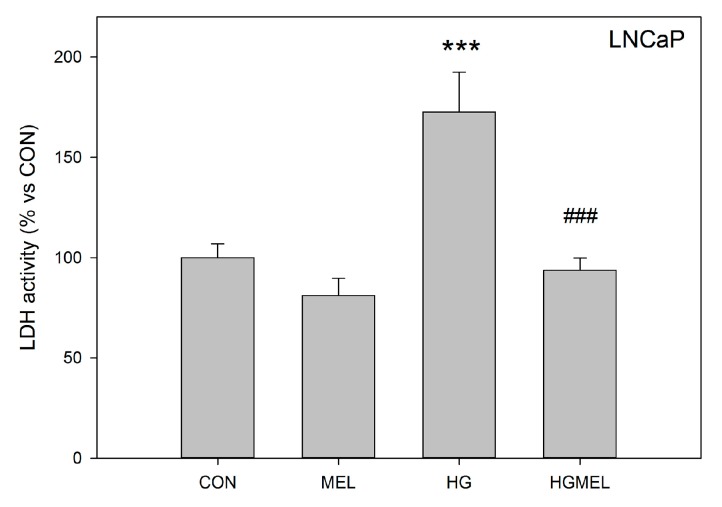
Melatonin decreases lactate dehydrogenase (LDH) activity in LNCaP cells. LDH activity was measured in LNCaP cells after grown in media supplemented with 2 g/L glucose (CON) or 4 g/L glucose (HG) with or without 1 mM melatonin (MEL) for 24 h. Results are shown as the percentage vs. CON group and a representative of three independent experiments is shown. Data are shown as mean ± SEM (*n* = 3). *** *p* < 0.001 vs. CON, ^###^ *p* < 0.01 vs. HG.

In order to corroborate the decrease of ^13^C-labeled lactate shown above, our next approach was to study LDH activity in melatonin-incubated cells. When LNCaP cells were incubated with melatonin in normal glucose conditions, only a slight non-significant decrease in LDH activity was found. On the other hand, LDH activity dramatically increased under HG culture conditions, but this increment was significantly reduced by melatonin (Figure 8).

### 2.6. Melatonin Reduces Glucose-6-Phosphate Dehydrogenase (G6PDH)

**Figure 9:**
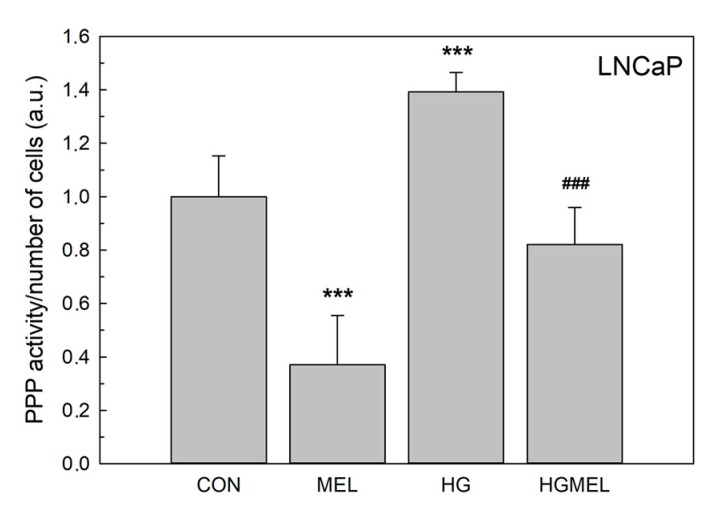

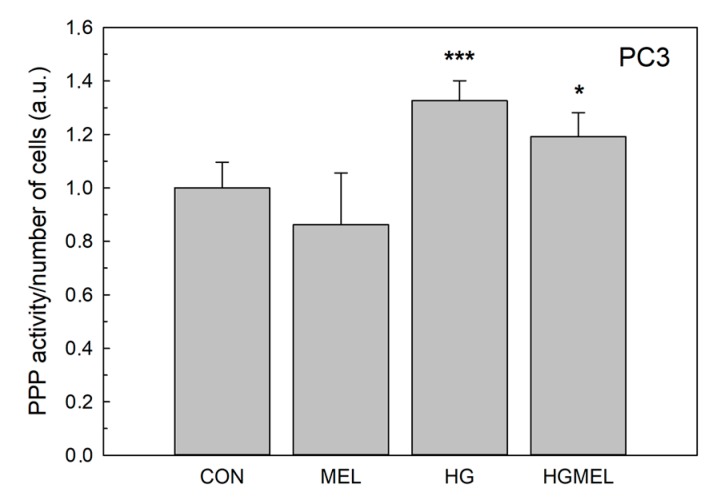
Melatonin reduces pentose phosphate pathway (PPP) in LNCaP and PC-3 cells. PPP activity measured as glucose-6-phosphate dehydrogenase (G6PDH) and 6-phosphogluconate dehydrogenase (6PGD) activity in LNCaP (**A**); and PC-3 cells (**B**) grown in media supplemented with 2 g/L glucose (CON) or 4 g/L glucose (HG) with or without 1 mM melatonin (MEL) for 24 h. Arbitrary 1.0 was given to CON groups. Results are shown as mean ± SEM (*n* = 3).* *p* < 0.05 vs. CON, *** *p* < 0.001 vs. CON, ^###^ *p* < 0.01 vs. HG.

Given that TCA did not change after HG culturing or after melatonin treatment in androgen-independent PC-3 cells, our next goal was to consider whether a potential glycolysis deviation toward the pentose phosphate pathway (PPP) could occur. To this aim, the levels of glucose-6-phosphate dehydrogenase (G6PDH), which is activated upon a higher demand of this biosynthetic pathway, was evaluated. Under normal glucose conditions, incubation with melatonin caused a dramatic reduction in the PPP activity in LNCaP cells (Figure 9). HG media induced a remarkable increase in G6PDH levels in both cell types. More importantly, melatonin totally limited the rise in PPP activity caused by HG in androgen-dependent cells, while there was no significant reduction in G6PDH in PC-3 cells.

## 3. Discussion

Melatonin has multiple activities in target cells. Some of them are mediated by its binding to its membrane receptors while others are receptor-independent or mediated by intracellular targets [35]. For years, we have wondered why melatonin needs pharmacologic concentrations to exert some of its biological functions, particularly when it is assayed in vitro. Several reports have proposed that melatonin easily crosses cell membranes, mainly due to its lipophilic nature and small size. However, it has been suggested that this fact does not properly explain the necessity of such a high concentration of the indole, specifically in cell culture studies. In this way, we have recently proved that melatonin crosses prostate cancer cells membranes, but using an active or facilitative transport [19]. This led us to discover facilitative glucose transporter 1 (GLUT1) as a potential carrier for melatonin in prostate cancer cells [22]. Melatonin uptake using GLUT1 transporter seemed to compete with glucose uptake itself, which could explain its antitumor properties. However, it has not been demonstrated yet melatonin’s role on oxidative phosphorylation (OXPHOS) or glycolysis in androgen dependent or independent prostate cancer cells.

The prostate is a particular gland from the metabolic point of view. Contrary to other tumors that highly increase glucose uptake early during transformation, prostate cancer cells depend on OXPHOS at the initial stages of the disease and glycolysis is only remarkable in the final steps of the illness. This metabolic phenomenon actually limits the use of diagnostic procedures such as PDG-PET scan for the detection of tumors at those early stages [36]. For this reason, we evaluate here the isotopic enrichment of mitochondrial metabolites after culturing cells with ^13^C-glucose labeled when melatonin is present in the culture media. In order to evaluate the role of the indole on glucose metabolism, the experimental approach followed included both normal (2 g/L) and high (4 g/L) glucose concentration scenarios. Interestingly, we found that melatonin reduces glucose uptake as previously demonstrated, but also reduces ATP production. In addition, the ATP/AMP ratio is higher in androgen sensitive LNCaP than in androgen insensitive PC-3 cells, which implies that PC-3 also represents a better model for an advanced stage of the disease from the metabolic point of view. In both cell types, melatonin reduces glucose uptake and the rise in ATP levels, thus indicating that it does not affect particularly at any stage, androgen sensitive or castration resistant phenotype. Interestingly, regarding CO_2_ production, differences were only detected when androgen sensitive prostate cancer cells were cultured in high-glucose concentration media. These results should be taken cautiously, due to the technical limitations. Thus, ^13^CO_2_ measurement, which requires a tight capping of culture flasks, cannot be performed in long-term experiments (more than 6 h). Otherwise, this would limit O_2_ concentration within the tissue culture flask and therefore compromise cell metabolism and survival. Clearly, the glucose increase caused a parallel rise in ^13^C-labeled CO_2_ but no significant differences were observed in melatonin treated cells.

In vitro androgen-sensitive cells represent the first stages of prostate cancer progression, which should imply that they favor glucose metabolism through OXPHOS. However, it was recently reported that androgens increase glycolytic metabolism in LNCaP cells based on an increment in glucose uptake, fosfofructokinase 1 activity, and lactate production [37]. From metabolomic studies showed here, we concluded that the enrichment in lactate decreased significantly in LNCaP cells after melatonin treatment, which it is in agreement with LDH activity, and it implies that melatonin decreases aerobic glycolysis. However, melatonin also reduces the isotopic enrichment of citrate, succinate, fumarate and malate, therefore indicating a slow-down in the tricarboxylic acid cycle (TCA) in androgen dependent prostate cancer cells. Finally, in our model melatonin also reduces the pentose phosphate pathway (PPP), represented by the increment of glucose-6-phosphate dehydrogenase (G6PDH). Altogether, these data clearly demonstrate that melatonin reduces all the major pathways of glucose metabolism and this can only be explained in terms of a general reduction in the glucose uptake in these cells.

According to the classic Warburg effect theory, differentiated cells obtain their energy from ATP through mitochondrial respiration and oxidative phosphorylation [38]. However, cancer cells are characterized by a high rate of glycolysis even under aerobic condition [39]. In one way, a high glycolysis rate in cancer cells satisfies a higher ATP demand and contributes to cell proliferation, survival and enhanced production of macromolecules such as lipids, proteins, and nucleic acids [40,41]. For this reason, glycolysis has been recently suggested as a major hallmark of invasive cancers [42] because in cancer cells, glycolysis contributes to metastasis. Nevertheless, this situation seems to be particularly different in prostate cancer cells [33,43]. In mitochondria from prostate epithelial cells, citrate oxidation is impaired and citrate is the final product of the glucose metabolism. However in cancer cells, citrate represents an efficient source of energy and they manage to restore oxidation through the Krebs cycle [44]. In reference to that, melatonin reduces citrate oxidation through TCA because glutamate and succinate showed significant differences in ^13^C labeling, not only when cultured in high glucose media but also when cells were cultured under low glucose conditions.

Interestingly, in PC-3 cells melatonin reduces the uptake of glucose and ATP production, being the levels of ATP in PC-3 cells even lower than in LNCaP. However, Krebs cycle did not change significantly after culturing cells in high glucose media nor after melatonin treatment, which points that TCA does not change in androgen independent prostate cancer cells. On the other hand, melatonin reduces lactate labeling suggesting that the indole should be able to reduce glycolysis also in these cells.

It has been shown that melatonin inhibits hypoxia-inducible factor 1α (HIF-1α) in vivo and in vitro [45]. The role of HIF-1α in cancer cells metabolism has been extensively demonstrated [46,47]. Among others, HIF-1α activates *SLC2A1* and *SLC2A3*, which encode the glucose transporters GLUT1 and GLUT3, respectively [48]. It would be feasible to think that melatonin through HIF-1α could modify GLUT1 expression, reducing the levels of the transporter and then the uptake of glucose. Although in another experimental models, the role of melatonin in glycolysis has been attributed to HIF-1α [45], this is not the case in our model. First, the reduced availability of O_2_ found in many solid tumors activates HIF-1α and its down-regulated pathways. Under normoxy conditions, HIF-1α has a short-life and it is stabilized when O_2_ concentrations are below 5%. The standard culture conditions, i.e., 5% CO_2_:95% air, assure a partial pressure of O_2_ of 150 mmHg or an equivalent of 20% O_2_. Then, HIF-1α was undetectable in LNCaP and PC-3 cells (data not shown). In prostate cancer, the principal regulator of GLUT1 expression is the AMP-activated protein kinase (AMPK) by an indirect mechanism that is further control by androgens [49]. In addition, HIF-1 sustains glycolytic metabolism, but LNCaP cells, as an example of initial stages of prostate cancer, shows a metabolism more oxidative than glycolytic, which is the principal target of melatonin.

Collectively, the effects of melatonin on glucose metabolism might mediate its role as an antitumor agent in prostate cancer cells. In fact, androgens increase lactate production and release through an increment in MCT4 transporter [37]. High LDH serum levels are associated with a poor survival in prostate cancer patients [50] and, consequently, the enzyme has been proposed target for future therapies in cancer [51].

Melatonin displays significant beneficial effects against hyperglycemia-induced cellular toxicity [52] and exerts different effects on glucose uptake by cells. In one hand, the indoleamine is able to restore insulin-induced glucose uptake in skeletal muscle cells [53] or increase the uptake in 3T3-L1 adipocytes [54] while on the other hand, it reduces glucose uptake in cancer cells [22]. A decrease in LDH activity has also been observed when cells were treated with melatonin and lipopolisaccaride (LPS) [55].

In general, melatonin reduces glucose uptake likely through the blocking of facilitative glucose transporters as it has been previously demonstrated [22,56]. GLUT1 is expressed in human prostate carcinoma cells [57] and it is upregulated by androgens in androgen sensitive prostate cancer cells [39,58]. In addition, the molecular characterization of Gleason 3 + 3 or 4 + 3 human prostate cancers, which greatly differ in patients outcome, might be related to reverse Warburg effect-associated genes, including GLUT1 [59]. An increase in GLUT1 and the resulting increase in lactate production has been related to the role of microRNA-132 knockdown of in prostate cancer progression [60].

## 4. Materials and Methods

### 4.1. Cell Culture

The human androgen-sensitive LNCaP prostate cell line was obtained from “European Collection of Cell Cultures” (cat^#^ 89110211 ECACC, Wiltshire, UK). Human androgen-insensitive PC-3 cells were obtained from “American Type Culture Collection” (cat^#^ CRL-1435 ATCC, Rockville, MD, USA). LNCaP cells were maintained in RPMI 1640 medium supplemented with 10% Fetal Bovine Serum (FBS), 2 mM l-glutamine (Gln), 15 mM HEPES and an antibiotic-antimycotic cocktail containing 100 U/mL penicillin, 10 µg/mL streptomycin and 0.25 µg/mL amphotericin B. PC-3 cells were grown in DMEM/F12 medium supplemented with 10% FBS, 2 mM l-Gln and the antibiotic-antimycotic cocktail described above. Both cell lines were grown at 37 °C in a humidified 5% CO_2_ atmosphere. The medium was changed every 2 days and cultures were split at least once a week. Melatonin was always freshly prepared in a DMSO stock solution 1000× and diluted to 1 mM concentration directly in the culture medium. DMSO 0.001% was always added to control groups. Toxicity of 0.001–0.01% of DMSO was tested in PCa cells prior our experiments.

### 4.2. Glucose Uptake

Glucose uptake was assessed as described previously [60] with minor modifications. Cells were seeded in 24-well culture plates and grown for 24 h in regular complete media with glucose. After washing cells twice with RPMI 1640 without glucose, medium containing 2 mM 2-deoxy-d-glucose (2-DG) was then added and cells were further incubated for 20 min at 37 °C in a 5% CO_2_ atmosphere. After that, cells were washed twice with KRH buffer (50 mM HEPES, 137 mM NaCl, 4.7 mM KCl, 1.85 mM CaCl_2_ and 1.3 mM MgSO_4_, pH 7.4) containing 0.1% of BSA (Cohn fraction V) and finally lysed in 0.1 M NaOH by heating samples from 60 to 85 °C for 60 min. After that, lysates were neutralized with 0.1 M HCl and the same volume of 200 mM triethanolamine (TEA) was added to each well. Twenty-five microliters were transferred to 96-well plates and 200 µL of assay solution (50 mM TEA, 50 mM KCl, 0.02% BSA, 0.1 mM NADP^+^, 0.2 U/mL diaphorase, 6 µM resazurin sodium salt and 20 U/mL glucose-6-phosphate dehydrogenase) were added to each well. Plates were incubated for 45 min at 37 °C. Finally, fluorescence was measured in a Sinergy H4 Hybrid Reader (λ_ex_ = 550 nm, λ_em_ = 605 nm). For standardization, protein concentration was determined using Bradford assay (BQCkit, Llanera, Spain). Results are presented as mmol 2-DG glucose/µg protein. Results are shown as the mean of three samples ± SEM.

For radioactive assays, cells were seeded in 6-well plates. After treatment, cells were harvested by trypsinization and counted using a Neubauer hemocytometer. In total, 25 × 10^5^ LNCaP cells and 15 × 10^4^ PC3 cells were employed. After centrifuged, cells were resuspended in 30 µL of RPMI without glucose and incubated for 30 min at 37 °C. Then, 2DG uptake was performed. 2DG uptake was initiated by the addition of labeled 2-deoxy-d-[1-^3^H]glucose to a final concentration of 0.5 µM (2 µCi) (1 Ci = 37 GBq). Incubation was prolonged for 10 min at room temperature. Cells were washed twice in PBS with 2% FBS and then, lysed with 0.5 mL 0.1% SDS. Finally, radioactivity was measured by liquid scintillation.

### 4.3. ATP Analysis

LNCaP and PC-3 cells were seeded at a density of 25,000 cell/mL. After 24 h, cells were scraped and centrifuged at 500× *g* at 4 °C. The cell pellet was dissolved in 0.3 M perchloric acid, gently mixed and centrifuged at 9000× *g* at 4 °C. Then, supernatants were neutralized with 1 M KOH, centrifuged at 9000× *g* and finally HPLC assay was performed. Adenine nucleotides (ATP, ADP and AMP) were separated on a 15 cm × 4.6 mm, 3 µm SUPELCOSIL LC-18-T column (Supelco, Sigma-Aldrich Quimica SL, Madrid, Spain) with a mobile phase composed of A mobile phase KH_2_PO_4_/tetrabutylammonium hydrogen sulfate (TBHS), pH 6.0 and B mobile phase MEOH + KH_2_PO_4_/TBHS, pH 5.5, at flow rate of 1.2 mL and in gradient mode: 0 min (0% B), 2.5 min (0% B), 7 min (20% B), 14 min (40% B), 19 min (100% B), 24 min (100% B), and 27 min (0% B). Nucleotides were detected by UV absorption at 260 nm, identified, and quantified by comparison with the retention time of appropriate standards.

### 4.4. Measurement of the ^13^CO_2_ Production in Cell Culture

LNCaP and PC-3 cells were seeded in a T25 flask at 25,000 cells/mL. After 48 h, media was changed and U-^13^C-d-glucose was added. For this set of experiments, flask caps were substituted by septum caps. The analysis of CO_2_ generated was performed at the beginning (*t* = 0) or after 6 h. A GC-IRMS instrument (Thermo Fisher Scientific, Bremen, Germany) was used for the determination of the ^13^C isotopic enrichment. The equipment consisted in a gas chromatograph with split/splitless injector coupled to an isotope ratio mass spectrometer. The mass analyzer is designed to measure CO_2_ isotope ratio with the highest precision and includes a magnetic sector to separate the ion beams for the ^12^CO_2_ and ^13^CO_2_ masses and an array of Faraday cups that allows the simultaneous measurement of both ion beam intensities. The Faraday cups have a different amplification in order to obtain comparable signals for the two CO_2_ isotopologues. Fifty microliters of the gas phase were withdrawn using a 1 mL gas-tight syringe and injected directly in the injection port of the chromatograph with a split ratio of 1:20. Helium, at a flow rate of 1 mL/min, was used as carrier gas. Measurements were run in triplicates.

### 4.5. Stable Isotope Labeling Experiments

For isotope labeling experiments, cells were cultured in 60 mm plates. When cells reached 70% confluence, the medium was replaced by glucose-free culture medium and U-^13^C-d-Glucose was added instead. After 48 h, cells were scraped and centrifuged at 500× *g* at 4 °C. Then, intracellular metabolites were obtained from previously pelleted cells after extraction with 100% methanol at −80 °C, following a double extraction with milli-Q water. Cell extracts were suspended in 500 µL of cold methanol, snap-freeze in liquid nitrogen and thawed in a mixture of liquid nitrogen-acetone. Later, extracts were vortexed for 30 s and pelleted by centrifugation at 800× *g* for 1 min. Supernatants were transferred to a fresh microcentrifuge tube in a liquid nitrogen-acetone mixture. They were again resuspended in 500 µL of methanol and freeze-thaw cycles were repeated. The supernatant was transferred and pooled with the previous methanol extraction. Finally, pellets were suspended in 250 µL of milli-Q water and freeze-thaw cycles were repeated for the last time. Cell extracts were then vortexed for 30 s and pelleted by centrifugation at 15,000× *g* for 1 min. Supernatants were removed and pooled with previous extracts. Pooled supernatant fractions were centrifuged at 15,000× *g* for 1 min in order to remove any cell debris remaining. Afterwards, supernatants were transferred to a fresh tube and dry at 30 °C in a vacuum centrifuge. Dried intracellular metabolites were kept at −80 °C prior derivatization for GC-MS analysis.

Metabolites extracted were dissolved in 100 µL of 2% methoxyamine hydrochloride in pyridine and incubated at 40 °C for 8 min on a compact thermomixer. Next, 150 µL of *N*-tert-butyldimethylsilyl-*N*-methyltrifluoroacetamide plus 1% tert-butyldimethylchlorosilane (TBDMCS) were added and samples were incubated for 10 min at 60 °C. Derivatized samples were centrifuged at 14,000× *g* for 2 min to remove debris. Clear samples were transferred into GC vials for GC-MS analysis. A gas chromatograph Agilent 7890 (Agilent, Santa Clara, CA, USA) coupled to a triple quadrupole mass spectrometer Agilent 7000 Series Triple Quad GC/MS (Agilent) was employed operating in selected-ion monitoring (SIM) mode at 70 eV. The GC was fitted with a split/splitless injector and a DB-5 MS capillary column (cross-linked 5% phenyl-methyl siloxane, 30 m × 0.25 mm i.d., 0.25 µm coating). The column temperature was initially set at 60 °C for 1 min, and then a temperature ramp of 5 °C/min was applied until it reached 320 °C for 10 min. The total run time was 68 min. Helium was used as carrier gas at a flow rate of 2 mL/min. The injector temperature was kept at 250 °C while interface temperature and ion sources were 280 °C and 230 °C respectively. Two microliters were injected in splitless with 1 min of purge time. Mass spectra were registered in a SIM mode using a 10 ms dwell time.

For the measurement of the mass isotopomer distribution of the intracellular metabolites, the experimental isotopic distribution measured by GC-MS were assumed to be a linear combination of all possible incorporation of ^13^C in the molecule (isotopomers). The relative contribution of isotope patterns in the experimental mass spectra is calculated by multiple linear regression. More details on this procedure are reported by Fernandez-Fernandez et al. [61]. Heat map and clustering analysis for data visualization of the identified metabolites were performed by the heatmap3 package in R project [62].

### 4.6. GA6PDH Activity

Glucose-6-phosphate dehydrogenase (G6PDH) and 6-phosphogluonate dehydrogenase (6PGDH) were measured as previously described with minor modifications [63]. Cells were grown in 24-well plates. After treatment, cells were incubated with 1/3 volume of 1 mM tetranitroblue tetrazolim, 0.5 mM glucose-6-phosphate, and 0.5 mM NADP^+^ for 4 h. The development of purple color was measured spectrophotometrically. For standardization, cell number was previously calculated using a Neubauer hemocytometer counting chamber. Results are shown as the mean of three samples ± SEM.

### 4.7. LDH Activity

For lactate dehydrogenase (LDH) assay, LNCaP cells were seeded in 6-well plates. After 24 h of treatment, total LDH activity was performed following manufacture’s specifications ref^#^ KC-04-004 BQCkit (BQC, Llanera, Spain). Absorbance was determined by using an automatic microplate reader Cary-50 (Agilent Technologies, Santa Clara, CA, USA) at 490 nm

### 4.8. Statistics

Unless otherwise indicated, all experiments were performed at least three times and each experimental group in triplicates. An ANOVA, followed by a Student Newman–Keuls *t*-test was performed and significant differences were considered when *p* < 0.05.

## 5. Conclusions

Metabolomics showed that melatonin decreases aerobic glycolysis in androgen sensitive prostate cancer cells. However, it also reduces the isotopic enrichment of citrate, succinate, fumarate and malate, therefore indicating a slow-down in tricarboxylic acid cycle (TCA) and glucose-6-phosphate dehydrogenase (G6PDH) activity suggesting a decrease in pentose phosphate pathway (PPP). Consequently, melatonin reduces all the major pathways of glucose metabolism of prostate cancer cells which implies that melatonin promotes a general reduction in the glucose uptake by these cells. Considering these results, it is reasonable to think that glucose transporters are direct targets for melatonin, which might mediate its antitumor properties not only in prostate cancer cells but also in other cancer cell types through a general reduction of glucose metabolism.

## References

[1] Stehle J.H., Saade A., Rawashdeh O., Ackermann K., Jilg A., Sebestény T., Maronde E. (2011). A survey of molecular details in the human pineal gland in the light of phylogeny, structure, function and chronobiological diseases. J. Pineal Res..

[2] Iriti M., Varoni E.M., Vitalini S. (2010). Melatonin in traditional Mediterranean diets. J. Pineal Res..

[3] Hardeland R., Poeggeler B. (2003). Non-vertebrate melatonin. J. Pineal Res..

[4] Singh M., Jadhav H.R. (2014). Melatonin: Functions and ligands. Drug Discov. Today.

[5] Tan D.-X., Hardeland R., Manchester L.C., Paredes S.D., Korkmaz A., Sainz R.M., Mayo J.C., Fuentes-Broto L., Reiter R.J. (2010). The changing biological roles of melatonin during evolution: From an antioxidant to signals of darkness, sexual selection and fitness. Biol. Rev. Camb. Philos. Soc..

[6] Reiter R.J., Mayo J.C., Tan D.-X., Sainz R.M., Alatorre-Jimenez M., Qin L. (2016). Melatonin as an antioxidant: Under promises but over delivers. J. Pineal Res..

[7] Ren W., Liu G., Chen S., Yin J., Wang J., Tan B., Wu G., Bazer F.W., Peng Y., Li T. (2017). Melatonin signaling in T cells: Functions and applications. J. Pineal Res..

[8] Sainz R.M., Mayo J.C., Rodriguez C., Tan D.X., Lopez-Burillo S., Reiter R.J. (2003). Melatonin and cell death: Differential actions on apoptosis in normal and cancer cells. Cell. Mol. Life Sci..

[9] Tan D., Reiter R.J., Chen L.D., Poeggeler B., Manchester L.C., Barlow-Walden L.R. (1994). Both physiological and pharmacological levels of melatonin reduce DNA adduct formation induced by the carcinogen safrole. Carcinogenesis.

[10] Sainz R.M., Mayo J.C., Tan D.-X., Lopez-Burillo S., Natarajan M., Reiter R.J. (2003). Antioxidant activity of melatonin in Chinese hamster ovarian cells: Changes in cellular proliferation and differentiation. Biochem. Biophys. Res. Commun..

[11] Boutin J.A., Audinot V., Ferry G., Delagrange P. (2005). Molecular tools to study melatonin pathways and actions. Trends Pharmacol. Sci..

[12] Erşahin C., Masana M.I., Dubocovich M.L. (2002). Constitutively active melatonin MT(1) receptors in male rat caudal arteries. Eur. J. Pharmacol..

[13] Tengattini S., Reiter R.J., Tan D.-X., Terron M.P., Rodella L.F., Rezzani R. (2008). Cardiovascular diseases: Protective effects of melatonin. J. Pineal Res..

[14] Tan D.-X., Manchester L.C., Fuentes-Broto L., Paredes S.D., Reiter R.J. (2011). Significance and application of melatonin in the regulation of brown adipose tissue metabolism: Relation to human obesity. Obes. Rev..

[15] Acuña Castroviejo D., López L.C., Escames G., López A., García J.A., Reiter R.J. (2011). Melatonin-mitochondria interplay in health and disease. Curr. Top. Med. Chem..

[16] Tan D.-X., Manchester L.C., Terron M.P., Flores L.J., Reiter R.J. (2007). One molecule, many derivatives: A never-ending interaction of melatonin with reactive oxygen and nitrogen species?. J. Pineal Res..

[17] Rodriguez C., Mayo J.C., Sainz R.M., Antolín I., Herrera F., Martín V., Reiter R.J. (2004). Regulation of antioxidant enzymes: A significant role for melatonin. J. Pineal Res..

[18] Korkmaz A., Topal T., Tan D.-X., Reiter R.J. (2009). Role of melatonin in metabolic regulation. Rev. Endocr. Metab. Disord..

[19] Hevia D., Sainz R.M., Blanco D., Quiros I., Tan D.-X., Rodríguez C., Mayo J.C. (2008). Melatonin uptake in prostate cancer cells: Intracellular transport versus simple passive diffusion. J. Pineal Res..

[20] Hevia D., González-Menéndez P., Quiros-González I., Miar A., Rodríguez-García A., Tan D.-X., Reiter R.J., Mayo J.C., Sainz R.M. (2015). Melatonin uptake through glucose transporters: A new target for melatonin inhibition of cancer. J. Pineal Res..

[21] Dauchy R.T., Hoffman A.E., Wren-Dail M.A., Hanifin J.P., Warfield B., Brainard G.C., Xiang S., Yuan L., Hill S.M., Belancio V.P. (2015). Daytime blue light enhances the nighttime circadian melatonin inhibition of human prostate cancer growth. Comp. Med..

[22] Bazwinsky-Wutschke I., Bieseke L., Mühlbauer E., Peschke E. (2014). Influence of melatonin receptor signalling on parameters involved in blood glucose regulation. J. Pineal Res..

[23] Scheer F.A.J.L., Hilton M.F., Mantzoros C.S., Shea S.A. (2009). Adverse metabolic and cardiovascular consequences of circadian misalignment. Proc. Natl. Acad. Sci. USA.

[24] Nishida S., Segawa T., Murai I., Nakagawa S. (2002). Long-term melatonin administration reduces hyperinsulinemia and improves the altered fatty-acid compositions in type 2 diabetic rats via the restoration of Delta-5 desaturase activity. J. Pineal Res..

[25] Nishida S., Sato R., Murai I., Nakagawa S. (2003). Effect of pinealectomy on plasma levels of insulin and leptin and on hepatic lipids in type 2 diabetic rats. J. Pineal Res..

[26] Espino J., Pariente J.A., Rodríguez A.B. (2011). Role of melatonin on diabetes-related metabolic disorders. World J. Diabetes.

[27] Rondanelli M., Faliva M.A., Perna S., Antoniello N. (2013). Update on the role of melatonin in the prevention of cancer tumorigenesis and in the management of cancer correlates, such as sleep-wake and mood disturbances: Review and remarks. Aging Clin. Exp. Res..

[28] Bouatia-Naji N., Bonnefond A., Cavalcanti-Proença C., Sparsø T., Holmkvist J., Marchand M., Delplanque J., Lobbens S., Rocheleau G., Durand E. (2009). A variant near MTNR1B is associated with increased fasting plasma glucose levels and type 2 diabetes risk. Nat. Genet..

[29] Sainz R.M., Mayo J.C., Tan D., León J., Manchester L., Reiter R.J. (2005). Melatonin reduces prostate cancer cell growth leading to neuroendocrine differentiation via a receptor and PKA independent mechanism. Prostate.

[30] Mayo J.C., Hevia D., Quiros-Gonzalez I., Rodriguez-Garcia A., Gonzalez-Menendez P., Cepas V., Gonzalez-Pola I., Sainz R.M. (2016). IGFBP3 and MAPK/ERK signaling mediates melatonin-induced antitumor activity in prostate cancer. J. Pineal Res..

[31] Hanahan D., Weinberg R.A. (2011). Hallmarks of cancer: The next generation. Cell.

[32] Pavlova N.N., Thompson C.B. (2016). The emerging hallmarks of cancer metabolism. Cell Metab..

[33] Cutruzzolà F., Giardina G., Marani M., Macone A., Paiardini A., Rinaldo S., Paone A. (2017). Glucose metabolism in the progression of prostate cancer. Front. Physiol..

[34] Acuña-Castroviejo D., Escames G., Venegas C., Díaz-Casado M.E., Lima-Cabello E., López L.C., Rosales-Corral S., Tan D.-X., Reiter R.J. (2014). Extrapineal melatonin: Sources, regulation, and potential functions. Cell Mol. Life Sci..

[35] Yuan L., Collins A.R., Dai J., Dubocovich M.L., Hill S.M. (2002). MT(1) melatonin receptor overexpression enhances the growth suppressive effect of melatonin in human breast cancer cells. Mol. Cell Endocrinol..

[36] Schöder H., Larson S.M. (2004). Positron emission tomography for prostate, bladder, and renal cancer. Semin. Nucl. Med..

[37] Vaz C.V., Marques R., Alves M.G., Oliveira P.F., Cavaco J.E., Maia C.J., Socorro S. (2016). Androgens enhance the glycolytic metabolism and lactate export in prostate cancer cells by modulating the expression of *GLUT1*, *GLUT3*, *PFK*, *LDH* and *MCT4* genes. J. Cancer Res. Clin. Oncol..

[38] Parks S.K., Chiche J., Pouysségur J. (2013). Disrupting proton dynamics and energy metabolism for cancer therapy. Nat. Rev. Cancer.

[39] Beckner M.E., Stracke M.L., Liotta L.A., Schiffmann E. (1990). Glycolysis as primary energy source in tumor cell chemotaxis. J. Natl. Cancer Inst..

[40] Buchakjian M.R., Kornbluth S. (2010). The engine driving the ship: Metabolic steering of cell proliferation and death. Nat. Rev. Mol. Cell Biol..

[41] Gordan J.D., Lal P., Dondeti V.R., Letrero R., Parekh K.N., Oquendo C.E., Greenberg R.A., Flaherty K.T., Rathmell W.K., Keith B. (2008). HIF-α effects on c-Myc distinguish two subtypes of sporadic VHL-deficient clear cell renal carcinoma. Cancer Cell.

[42] Gatenby R.A., Gillies R.J. (2004). Why do cancers have high aerobic glycolysis?. Nat. Rev. Cancer.

[43] Singh K.K., Desouki M.M., Franklin R.B., Costello L.C. (2006). Mitochondrial aconitase and citrate metabolism in malignant and nonmalignant human prostate tissues. Mol. Cancer.

[44] Costello L.C., Franklin R.B. (2016). A comprehensive review of the role of zinc in normal prostate function and metabolism; and its implications in prostate cancer. Arch. Biochem. Biophys..

[45] Vriend J., Reiter R.J. (2016). Melatonin and the von Hippel-Linday/HIF-1 oxygen sensing mechanism: A review. Biochim. Biophys. Acta.

[46] Semenza G.L. (2013). HIF-1 mediates metabolic responses to intratumoral hypoxia and oncogenic mutations. J. Clin. Investig..

[47] Semenza G.L. (2017). Hypoxia-inducible factors: Coupling glucose metabolism and redox regulation with induction of the breast cancer stem cell phenotype. EMBO J..

[48] Barron C.C., Bilan P.J., Tsakiridis T., Tsiani E. (2016). Facilitative glucose transporters: Implications for cancer detection, prognosis and treatment. Metabolism.

[49] Frigo D.E., Howe M.K., Wittmann B.M., Brunner A.M., Cushman I., Wang Q., Brown M., Means A.R., McDonnell D.P. (2011). CaM kinase kinase β-mediated activation of the growth regulatory kinase AMPK is required for androgen-dependent migration of prostate cancer cells. Cancer Res..

[50] Petrelli F., Cabiddu M., Coinu A., Borgonovo K., Ghilardi M., Lonati V., Barni S. (2015). Prognostic role of lactate dehydrogenase in solid tumors: A systematic review and meta-analysis of 76 studies. Acta Oncol..

[51] Fiume L., Manerba M., Vettraino M., di Stefano G. (2014). Inhibition of lactate dehydrogenase activity as an approach to cancer therapy. Future Med. Chem..

[52] Jiang T., Chang Q., Zhao Z., Yan S., Wang L., Cai J., Xu G. (2012). Melatonin-mediated cytoprotection against hyperglycemic injury in Müller cells. PLoS ONE.

[53] Sartori C., Dessen P., Mathieu C., Monney A., Bloch J., Nicod P., Scherrer U., Duplain H. (2009). Melatonin improves glucose homeostasis and endothelial vascular function in high-fat diet-fed insulin-resistant mice. Endocrinology.

[54] Kato H., Tanaka G., Masuda S., Ogasawara J., Sakurai T., Kizaki T., Ohno H., Izawa T. (2015). Melatonin promotes adipogenesis and mitochondrial biogenesis in 3T3-L1 preadipocytes. J. Pineal Res..

[55] Song J., Kang S.M., Lee K.M., Lee J.E. (2015). The protective effect of melatonin on neural stem cell against LPS-induced inflammation. BioMed Res. Int..

[56] Wu H., Song C., Zhang J., Zhao J., Fu B., Mao T., Zhang Y. (2017). Melatonin-mediated upregulation of GLUT1 blocks exit from pluripotency by increasing the uptake of oxidized vitamin C in mouse embryonic stem cells. FASEB J..

[57] Chandler J.D., Williams E.D., Slavin J.L., Best J.D., Rogers S. (2003). Expression and localization of GLUT1 and GLUT12 in prostate carcinoma. Cancer.

[58] Gonzalez-Menendez P., Hevia D., Rodriguez-Garcia A., Mayo J.C., Sainz R.M. (2014). Regulation of GLUT transporters by flavonoids in androgen-sensitive and -insensitive prostate cancer cells. Endocrinology.

[59] Georgescu I., Gooding R.J., Doiron R.C., Day A., Selvarajah S., Davidson C., Berman D.M., Park P.C. (2016). Molecular characterization of Gleason patterns 3 and 4 prostate cancer using reverse Warburg effect-associated genes. Cancer Metab..

[60] Qu W., Ding S., Cao G., Wang S., Zheng X., Li G. (2016). MiR-132 mediates a metabolic shift in prostate cancer cells by targeting Glut1. FEBS Open Bio.

[61] Fernandez-Fernandez M., Rodriguez-Gonzalez P., Hevia D., Gonzalez-Menendez P., Sainz R.M., Garcia Alonso J.I. (2017). Accurate and sensitive determination of molar fractions of ^13^C-Labeled intracellular metabolites in cell cultures grown in the presence of isotopically-labeled glucose. Anal. Chim. Acta.

[62] Zhao S., Guo Y., Sheng Q., Shyr Y. (2014). Advanced heat map and clustering analysis using heatmap3. BioMed Res. Int..

[63] Tian W.N., Braunstein L.D., Apse K., Pang J., Rose M., Tian X., Stanton R.C. (1999). Importance of glucose-6-phosphate dehydrogenase activity in cell death. Am. J. Physiol..

